# Mucinous adenocarcinoma of a tailgut cyst

**DOI:** 10.4314/gmj.v56i1.8

**Published:** 2022-03

**Authors:** Philemon K Kumassah, Antoinette A A Bediako-Bowan, Nelson Agboadoh, Yaw B Mensah, Jonathan CB Dakubo

**Affiliations:** 1 Department of Surgery, Korle Bu Teaching Hospital, Accra, Ghana; 2 Department of Surgery, University of Ghana Medical School, College of Health Sciences, Accra, Ghana; 3 Department of Radiology, University of Ghana Medical School, College of Health Sciences, Accra, Ghana

**Keywords:** tailgut cyst, retrorectal hamartoma, presacral, mucinous, adenocarcinoma

## Abstract

A tailgut cyst is a rare congenital lesion that develops from a residual posterior remnant of the intestine and presents as a mass in the presacral space. They are generally asymptomatic or have atypical symptoms, are usually benign but may rarely become malignant. We report a case of a 37-year-old female who initially presented to the Surgical Department of Korle Bu Teaching Hospital, Accra, Ghana with a malignant tailgut cyst after having repeated surgical procedures for recurrent perianal infective pathologies but still had persistence of symptoms. The lesion was initially excised and found to be a dermoid cyst histologically. The mass recurred after a year and had a re-excision; the lesion was diagnosed histologically as mucinous adenocarcinoma. This report emphasizes the different forms of presentation of a patient with a tailgut cyst and the possibility of malignant transformation, as well as the presentation of this tailgut cyst which can be diagnosed using radiological investigations and histological findings.

**Funding:**

None

## Introduction

Retrorectal cystic hamartoma, or a tailgut cyst, is a rare congenital lesion that develops from a residual posterior remnant of the intestine and presents as a mass in the presacral space.^1^ The presacral (retrorectal) space is defined by the space bounded by the rectum anteriorly, the sacrum posteriorly, and peritoneal reflection superiorly (at the level of the junction of the second and third sacral segments), levator ani and coccygeus muscles inferiorly. The ureters and iliac vessels are the lateral margins of this space.^2^ In the early stages of development, the embryo possesses a tail, and the anus is formed cephalad to the tail. The primitive hindgut extends into the tail beyond the point at which the anus develops, hence the name tail-gut. Failure of regression of the hindgut leaves remnants of the tailgut, which can give rise to congenital cysts.^2^

Tailgut cysts are generally asymptomatic or may present with atypical symptoms resulting in delayed diagnosis.^3^ These cysts are usually benign but may rarely become malignant. The most common types of malignant transformations in tailgut cysts are adenocarcinoma, neuroendocrine tumour and squamous carcinoma.^4^ We report a case of a tailgut cyst that initially presented as a recurrent perianal abscess which was later excised and histologically diagnosed as a dermoid cyst.

The lesion recurred two years later and, upon adequate exposure and complete excision was found to have undergone a malignant transformation into a mucinous adenocarcinoma.

## Case Report

A 37-year-old lady presented with a year's history of change in bowel habit towards constipation, a feeling of a mass in the anal region and a feeling of incomplete emptying of her bowel. She neither had bleeding per rectum nor urinary symptoms and had not lost weight. Two years before the index presentation, an abdominopelvic magnetic resonance imaging (MRI) scan for similar complaints showed a huge multilobulated tailgut cyst. The patient had a laparotomy with excision of the cyst, and subsequent histology revealed a dermoid cyst. Symptoms, however, recurred a year after the excision of the dermoid cyst.

She also had a history of two surgical incisions and drainages performed for a recurrent perianal abscess, a month apart, a fistulectomy for a fistula-in-ano which developed as a complication from the incision and drainage. Rectal examination at index presentation revealed a firm, non-tender bulging mass in the posterior rectal wall with a mobile rectal mucosa over it. There was a significant narrowing of the rectal lumen.

**Figure 1:**
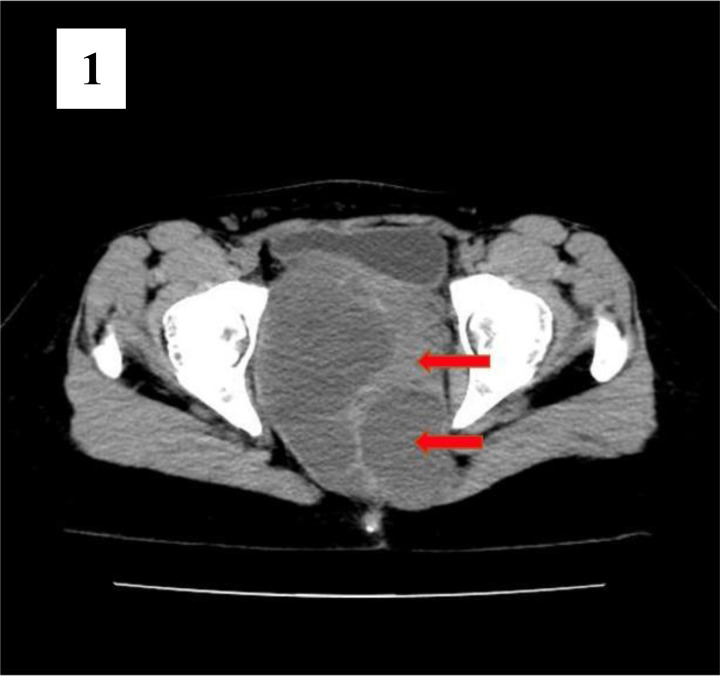
is an axial view of the non-contrast CT scan that shows both cystic and solid areas (red arrows).

**Figure 2:**
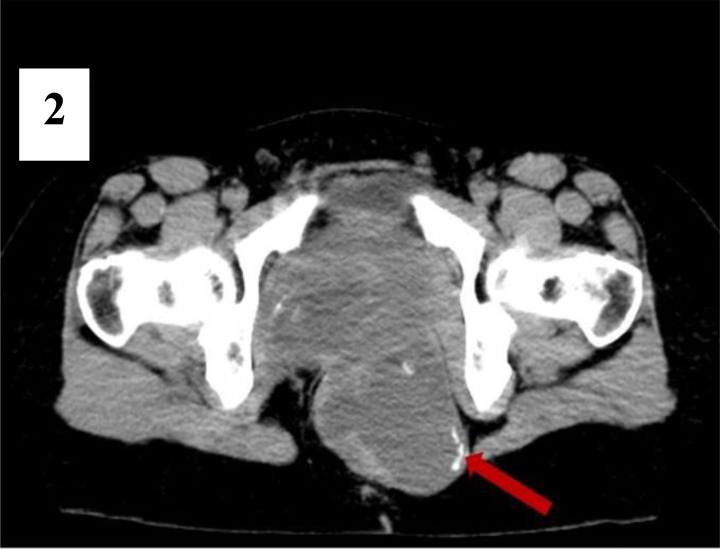
is an axial view of the non-contrast CT scan in which the periphery of the cystic component shows foci of coarse calcification (red arrow).

**Figure 3:**
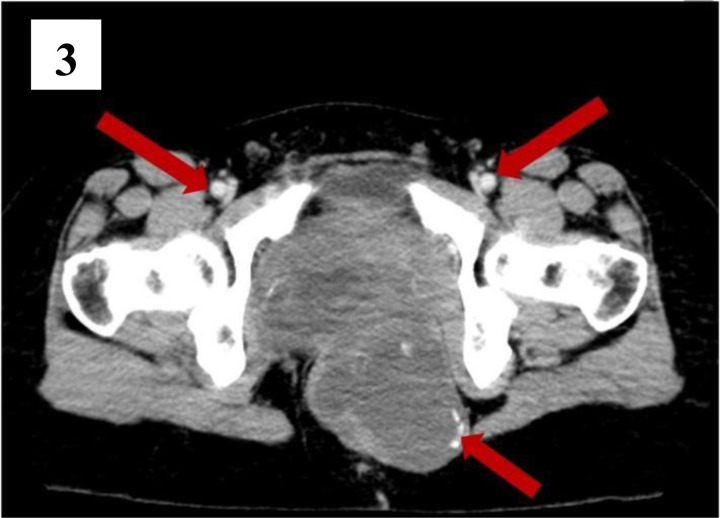
is an axial view of the contrast CT scan showing the contrast enhanced vessels (red arrows above the image) and foci of coarse calcification in the periphery of the cystic component (red arrow below the image).

**Figure 4:**
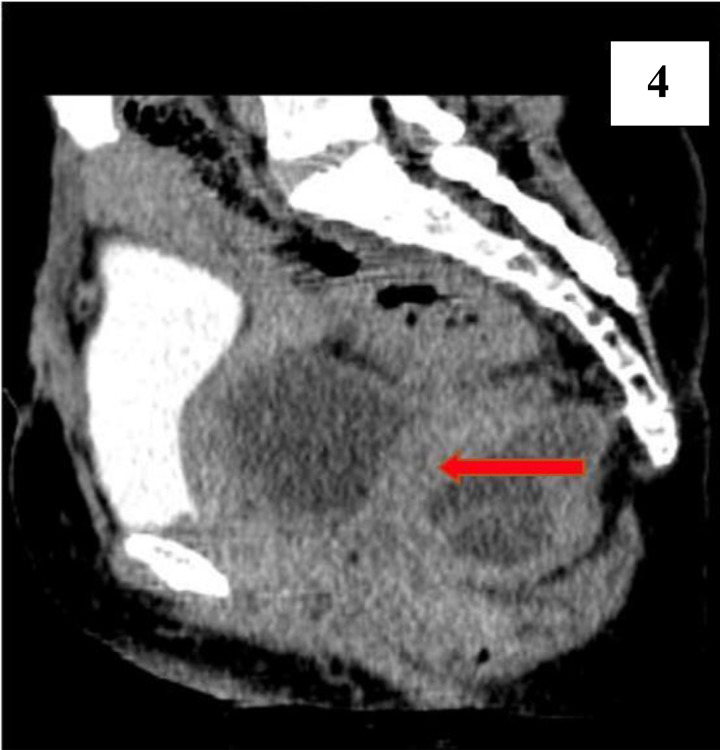
is a sagittal contrast view that clearly shows a 12mm septum between the cystic components (arrowed).

**Figure 5:**
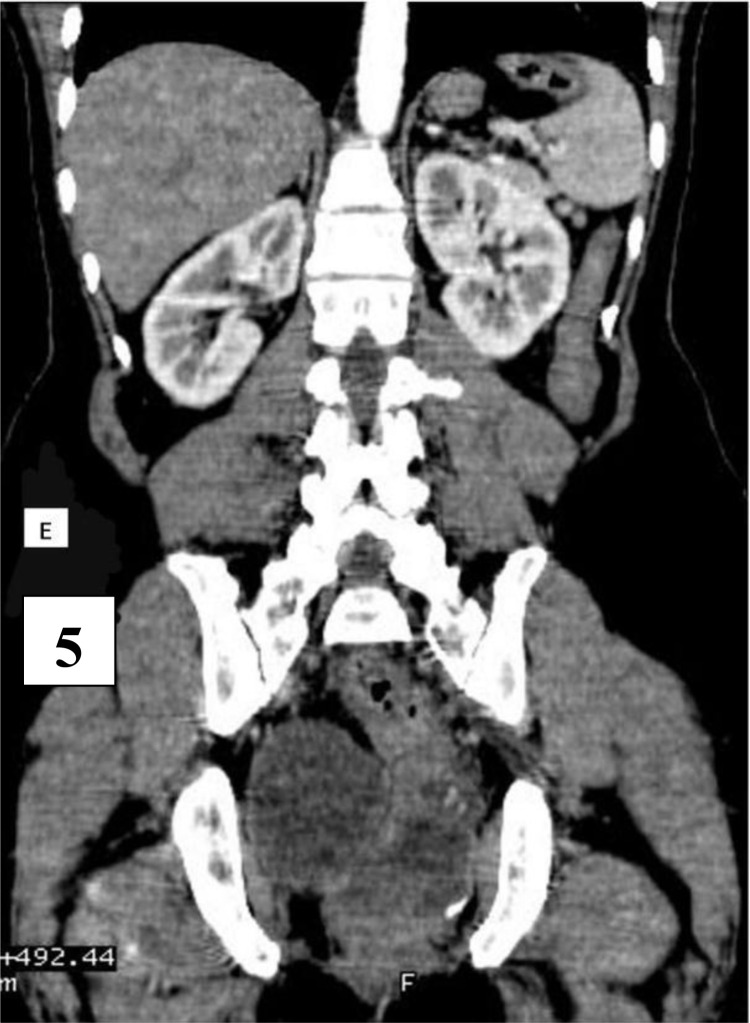
is the coronal contrast view showing the solid and cystic areas of the tailgut cyst

An abdominopelvic computer tomography (CT) scan showed a multilobulated presacral cystic lesion posterior to the anorectum (Figure 1) causing narrowing of the anorectal lumen with internal debris and some calcifications (Figures 2, 3, 4, 5). The CT scan also showed infiltration of adjacent obturator internus muscle.

She had an elevated serum carcino-embryonic antigen (CEA) of 37.9µg/L (normal range 0 -3µg/L). At surgery, a mass was found in the presacral region, posterior to the rectum, extending from the middle third down to the distal third of the rectum. The mass consisted of multiple presacral cysts containing myxomatous material.

The mass could not be excised en-bloc due to its attachment to the sacrum resulting in fragmentation of the mass.

A synchronous abdomino-transanal intersphincteric resection of the rectum was done, allowing adequate visualization of the mass within the presacral region. The mass was excised, followed by a coloanal anastomosis. Her immediate post-operative period was uneventful and was discharged on the sixth day after surgery.

**Figure 6:**
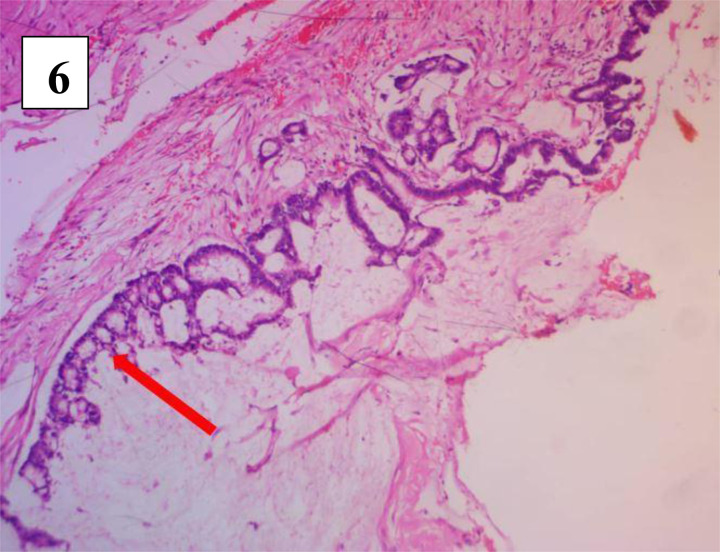
Microscopy showing multilocular cysts lined by mucinous epithelium

**Figure 7:**
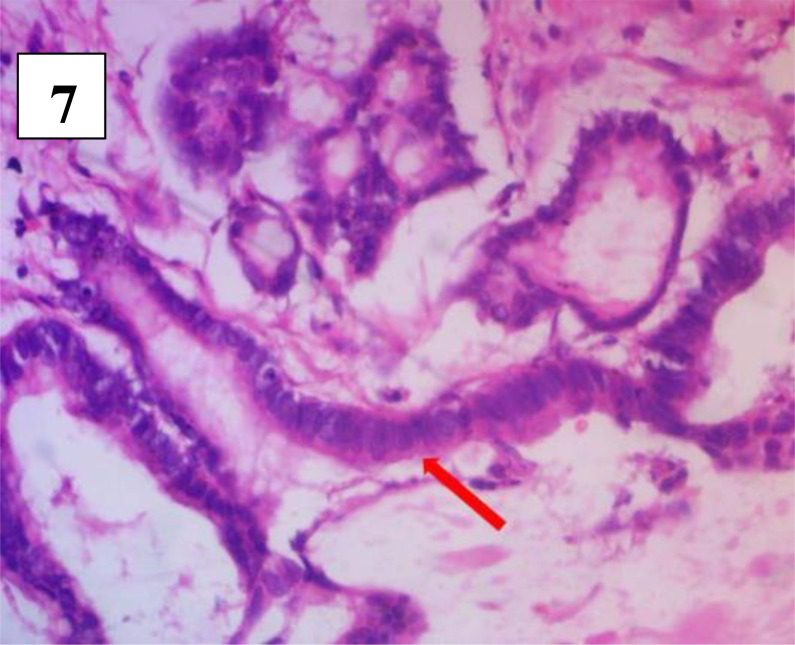
Microscopy showing Columnar epithelium with increased nuclear cytoplasmic ratios and hyperchromasi

The histopathology of the excised mass was a retro-rectal cystic harmatoma (tailgut cyst), with mucinous adenocarcinoma, showing no involvement/spread to the recto-anal wall. This was seen on microscopy as fragments of multiple cysts lined by both stratified squamous non-keratin-izing epithelium and simple columnar mucin secreting epithelium (Figure 6). The wall was composed of dense bundles of smooth muscle fibres, skeletal muscle fibres and lobules of mature adipocytes all interspersed by vascularized fibrocollagenous tissues. There were multiple foci of mucin collection with focal nuclear pleomorphism, hyperchromasia, loss of polarity and mitotic figures (Figure 7). She was referred to the radiation oncologists for adjuvant radiotherapy.

## Discussion

Tailgut cysts (TGC) are thin-walled, often multi-chamber, branching structures that rarely undergo malignant transformation. Malignant transformation is related to a gene mutation responsible for inhibiting the transformation of the p53 gene.^1^

Tailgut cysts may be confused with other pathologies in the presacral region, such as epidermal cysts, dermoid cysts, duplication cysts or teratomas,^5^ thus, one needs a high index of suspicion backed by good physical examination and cross-sectional imaging to make a diagnosis. Histology is usually needed to confirm the diagnosis.

This was evident in this patient who had several surgical procedures for other pathologies such as perianal abscess and fistula-in-ano other than a tailgut cyst. Though they can be found in all age groups, they commonly present in middle-aged females.^2^

Most lesions are asymptomatic and often discovered incidentally.^1^ Non-specific symptoms may occur from the pressure effect of the mass on adjacent structures,^1^ including difficulty in defecation, which this patient presented with. Other symptoms such as a change in stool calibre, perianal pain, urinary frequency and urinary retention may also be present.^1^

Tailgut cyst may also become infected and be misdiagnosed as a perianal abscess or anorectal fistula1 as was the case of this patient who had two surgeries on account of recurrent perianal abscess. The surgeries were further complicated by fistula-in-ano for which she had a fistulectomy. A carefully performed rectal examination can reveal a bulging mass through the posterior rectal wall, and that may give a clue to investigate the mass further.

Pelvic ultrasound, CT scan and MRI are valuable imaging modalities that aid in diagnosing tailgut cysts. Ultrasonography shows a complex mass not characteristic of a simple cyst with low-level internal echoes. The internal echoes of the tailgut cyst appear to result from the multicystic nature of the mass and the presence of keratinous material or inflammatory debris within the lumen.^6^ CT scan shows TGC as a well-defined retrorectal mass with the CT values ranging from water to soft-tissue density, as was the case of this patient's initial imaging. Malignant degeneration of a TGC may show as a loss of discrete margins, calcification inside the lesion and involvement of adjacent structures on CT scan.^6^ This was similar to what was noted on the patient's CT scan, which showed peripheral calcification, infiltration of adjacent structures and internal debris within the cystic lesion. Calcification is not common in TGC and its presence should raise the suspicion of a possible teratoma or malignancy^5^ which in the case of this patient was an adenocarcinoma.

MRI of a tailgut cyst may show as a lesion which is hypointense on T1W and hyperintense on T2W^4^ when uncomplicated which was the case at the time the patient had the MRI. However, when complicated it may be hyperintense on T1W.^4^ The MRI finding of a tailgut cyst in this patient was at variance with the histological finding of a dermoid cyst in the specimen from the initial excision. A request for review of pathological findings based on the MRI findings may help reconcile such discrepancies in the future.

Tailgut cysts which undergo malignant transformation to become adenocarcinoma may express carcinoembryonic antigen (CEA) and carbohydrate antigen 19-9 (CA19-9).^4^ This patient's CEA was as high as 37.9µg/L at presentation. The adenocarcinomas may also show evidence of upregulation of the expression of Ki-67 and p53.^4^ The level of CEA in these patients have been used as a marker to assess tumour response to treatment and for surveillance for recurrence.^5^ CA 19-9 and Ki-67 were not assessed for this patient.

Contrary to the usual trend of events, a percutaneous biopsy may fail to confirm a malignant diagnosis due to missing the localization. This, coupled with the fact that it carries a significant risk of spillage of malignant cells into the peritoneal cavity and seeding of malignant cells along the biopsy tract, the preoperative biopsy is not always recommended.^7^ In the setting of a heterogeneous mass with CEA elevation, a malignant process should therefore be assumed, and a biopsy avoided.^7^

Postsurgical resection histopathology remains the “gold standard” to diagnose TGC.^8^ In the patient presented, the diagnosis of mucinous adenocarcinoma of a tailgut cyst was made from the histology of the resected specimen. Microscopically, the lesions are usually multicystic and are lined by various epithelial cells commonly found in the adult and fetal gastrointestinal tract, including columnar, stratified columnar, transitional and squamous epithelia. Smooth muscle tissue bundles are almost always present in the cysts, but the muscle bundles are often disorganized and do not possess a myenteric plexus.^2^,^5^ In this patient, microscopy revealed multiple cysts lined by stratified squamous non-keratinizing and simple columnar epithelium and the wall composed of dense bundles of smooth and skeletal muscle fibres similar to what is documented in the literature.

Surgery is the treatment of choice for tailgut cysts. The principles guiding the surgery are a complete removal of the cyst with free margins; avoid rupture of cyst or cutting through tumor; and infiltrated structures should be resected en-bloc.^3^,^8^ In this patient, the tumour was entered into during the resection of the cyst and hence the exudation of myxomatous substance during the surgical procedure. This increases the chances of recurrence hence the patient is being keenly followed up for any sign of recurrence. The recurrence of the cyst after the first dermoid cyst excision could be due to an incompletely excised cyst wall.

There are four accepted surgical approaches to the tailgut cyst, including the sacrococcygeal approach, the single abdominal approach, the combined sacrococcygeal approach, and the anal approach. The sacrococcygeal approach is preferred for tumours at the level S3 or below and if there is bone involvement, while the abdominal approach is preferred for lesions above S3.^4^ If a malignant process is proven or suspected, the combined anterior laparotomy/posterior pelvic approach is recommended,^7^ which was the approach used in the patient presented.

For tailgut cysts with adenocarcinoma or squamous carcinoma transformation, adjuvant radiotherapy and chemotherapy are given after surgical resection.^4^,^8^ This patient also had a compounding factor of fragmentation of the mass at surgery; hence is currently undergoing chemotherapy to be followed by radiotherapy.

## Conclusion

Tailgut cysts should be considered in patients presenting with perianal pathologies such as presacral masses and features of malignant transformation sought with appropriate imaging studies. A thorough evaluation for perianal pathologies will enable prompt diagnosis and adequate surgical management.
